# The Slump Flow of Cementitious Pastes: Simulation vs. Experiments

**DOI:** 10.3390/ma17020532

**Published:** 2024-01-22

**Authors:** Mareike Thiedeitz, Thomas Kränkel, Deniz Kartal, Jithender J. Timothy

**Affiliations:** Centre for Building Materials, Department of Materials Engineering, TUM School of Engineering and Design, Technical University of Munich, 81245 München, Germany; mareike.thiedeitz@tum.de (M.T.);

**Keywords:** fresh cement paste flow, CFD, non-Newtonian flow, rheology, regularization, OpenFOAM

## Abstract

Understanding the transient properties of cementitious pastes is crucial for construction materials engineering. Computational modeling, particularly through Computational Fluid Dynamics (CFD), offers a promising avenue to enhance our understanding of these properties. However, there are several numerical uncertainties that affect the accuracy of the simulations using CFD. This study focuses on evaluating the accuracy of CFD simulations in replicating slump flow tests for cementitious pastes by determining the impact of the numerical setup on the simulation accuracy and evaluates the transient, viscosity-dependent flows for different viscous pastes. Rheological input parameters were sourced from rheometric tests and Herschel–Bulkley regression of flow curves. We assessed spatial and temporal convergence and compared two regularization methods for the rheological model. Our findings reveal that temporal and spatial refinements significantly affected the final test results. Adjustments in simulation setups effectively reduced computational errors to less than four percent compared to experimental outcomes. The Papanastasiou regularization was found to be more accurate than the bi-viscosity model. Employing a slice geometry, rather than a full three-dimensional cone mesh, led to accurate results with decreased computational costs. The analysis of transient flow properties revealed the effect of the paste viscosity on the time- and shear-dependent flow progress. The study provides an enhanced understanding of transient flow patterns in cementitious pastes and presents a refined CFD model for simulating slump flow tests. These advancements contribute to improving the accuracy and efficiency of computational analyses in the field of cement and concrete flow, offering a benchmark for prospective analysis of transient flow cases.

## 1. Introduction

In recent years, computational modeling of concrete casting using Computational Fluid Dynamics (CFD) has proven to be a suitable tool to estimate the processing properties (e.g., Wallevik and Wallevik in [1]) or to adjust concrete mixtures depending on the desired flow behavior without the need of experimental flow tests (e.g., de Schutter et al. in [2]). Experimental flowability analysis is possible using experimental rheometry (comprehensive overviews for cement and concrete, e.g., in [3,4,5,6]) or workability tests. The most common workability tests are the spread flow test, the V-funnel test, the slump test, the slump flow test, and the L-Box test. These tests analyze the workability of cementitious materials fast and in situ at construction sites and are also standardized to estimate rheological properties; see, e.g., [7,8,9]. Empirical formulas correlate flow test results to the yield stress $\tau_{0}$ (in Pa) and plastic viscosity μ or apparent viscosity η (in Pa∗s), as described by Roussel et al. in [10,11] for the slump flow test or Nguyen et al. in [12] for the L-Box test. All empirical correlations describe concrete as a viscoplastic fluid with a yield stress, which has to be surpassed before the concrete starts to flow. The correlation between the yield stress $\tau_{0}$ and the final radius of a slump flow measurement after stoppage of flow is [10]:

(1) $$\tau_{0A,R}\approx \frac{225\rho_{p}gV^{2}}{128\pi^{2}R^{5}}$$

where $\tau_{0,A.R}$ is the analytical yield stress in Pa, $\rho_{p}$ the density of the tested material in $kg/m^{3}$, V the tested volume in $m^{3}$, and R the final slump flow radius in m. Yield stress analysis with Equation (1) correlates with the rheological analysis from rheometric measurements for cement pastes with slump flow values >200 mm; see e.g., [13,14]. The L-Box test and slump flow test are also commonly used as model setups in computational models. By comparing the experimental measurements to the numerical result, the accuracy of the simulation can be estimated (e.g., [15,16,17]), or different simulation programs can be compared (e.g., [18]). Replicating the numerical slump flow test from the experimental test, then, becomes a feasible method to set up CFD models that can be used for the flow analysis of concrete processing scenarios. However, a closer look reveals drawbacks of the numerical L-Box or slump flow test case. “Ideal benchmark scenarios” compare fully resolved analytical solutions, e.g., for the velocity field, with the numerical approximation, and, thus, optimize the numerical setup toward a defined mathematical result. For certain geometries, an analytical solution exists, such as pipe flow (see benchmark application in [19]). In flow processes like debris flow, avalanches, or the slump flow test, calculating full velocity profiles analytically is, due to the transient flow, impossible. Both the flow field and material properties change constantly. Consequently, the verification of a numerical solution for the slump flow test is not straightforward, and a defined verification procedure does not exist [20]. Therefore, according to Frigaard and Nouar, the slump flow test is a “worst case scenario” [21]. More specifically, it has following numerical challenges:

(a) *Free surface flow*: The numerical CFD test case needs to define both suspension and air properties, as well as boundary conditions between walls/suspension, walls/air, and air/suspension. A slip condition between concrete and walls can be defined but affects the solution in a way that is difficult to prove.
(b) *Non-defined start-up of flow properties*: In an experimental test case, paste is filled into a cone and the cone is lifted [22]. While the lifting velocity has an effect on suspension properties, its real value is unknown, and the numerical implementation becomes complicated.
(c) *Spatial-temporally dependent transient flow conditions*: An accurate CFD simulation requires a numerically refined mesh specified according to the transport properties [23]. Non-dimensional numbers characterize the flow, such as the Courant number (Co). With time-dependent progressing flow characteristics, optimal mesh conditions can change.
(d) *Transition from flow to stoppage*: CFD is defined for flowing processes. A resting case with the velocity u=0 is numerically not defined. This yields two difficulties: First, a numerical regularization needs to be found for the transition toward velocities →0. Secondly, a threshold needs to define the numerical final flow length.

The evaluation of an accurate numerical test setup, however, is of utmost importance for prospective advancements in the field of computational concrete rheology. With the constant development of innovative concrete mixtures, such as Self-Compacting Concrete (SCC), Ultra-High-Performance Concrete (UHPC), concrete for Additive Manufacturing (AM), or concretes with low clinker amounts for the purpose of more ecological concrete mixtures, their rheological properties become increasingly complex toward higher viscosity values and strongly non-Newtonian flow behavior. While CFD offers a method for the precise computation of various processing scenarios without the need of expensive experimental tests, both the numerical setup and the rheological model need to be verified regarding their accuracy. Research on this topic is yet to be published. In this study, the effect of spatial-temporal meshing and the numerical regularization is analyzed on the model accuracy. Subsequently, the effect of rheological properties of increasingly viscous cementitious pastes is analyzed on the transient flow in a cementitious slump flow test setup.

## 2. Simulating Flow of Cementitious Pastes with Multiphase CFD Modeling

Cementitious pastes are rheologically described as non-Newtonian fluids with a yield stress. While various phenomenological equations have been proposed (comprehensive overviews, e.g., in [24,25,26]), the Bingham model is the most basic formulation [27]:

(2) $$\tau (\dot{\gamma })=\tau_{0,B}+\mu \dot{\gamma }$$

With $\tau \left(\dot{\gamma }\right)$ as the shear-rate dependent shear stress in Pa, $\tau_{0,B}$ as the Bingham—yield stress in Pa, $\dot{\gamma }$ as the shear rate in 1/s, and μ as the plastic viscosity in Pa∗s. The Herschel–Bulkley model in Equation (3) incorporates a non-linear viscosity [28]:

(3) $$\eta \left(\dot{\gamma }\right)=\frac{\tau_{0,H-B}}{\dot{\gamma }}+k{\dot{\gamma }}^{n-1}$$

With $\eta \left(\dot{\gamma }\right)$ as the shear-rate dependent apparent viscosity in Pa∗s, k as the consistency index in ${\left(Pa*s\right)}^{n}$, and n as the Herschel–Bulkley index, characterizing shear-thinning flow in case of n<1, Bingham flow in case of n=1, and shear-thickening flow if n>1.

CFD provides numerical algorithms to approximate the transport equations for viscous flow over space and time. The Volume-of-Fluid method (VOF) is a numerical technique to compute the free-surface flow of cementitious pastes. The Navier–Stokes transport equations, consisting of the equations for the conservation of mass and conservation of momentum, are extended by a weighting quantity α; see Equations (4) and (5), also applied in [1]:

(4) $$\frac{\delta \alpha }{\delta t}+\nabla \cdot (\alpha u)=0$$

(5) $$\frac{\partial (\rho u)}{\partial t}+\nabla \cdot (\rho uu)=\nabla p+\nabla \cdot \tau +\rho g+f_{\sigma }$$

In Equation (5), ρ is the fluid density in $kg/m^{3}$, u is the velocity vector in m/s, p is the scalar pressure in Pa, τ is the deviatoric stress tensor in Pa, g is the gravitational acceleration in $m/s^{2}$, and $f_{\sigma }$ is the contribution from surface tension effects between the two phases [29]. The fluid density ρ is calculated from the contributions of the two phases $\rho_{1}$ and $\rho_{2}$ depending on α:

(6) $$\rho =\alpha \rho_{1}+\left(1-\alpha \right)\rho_{2}$$

The deviatoric stress tensor τ contains the rheological model of the fluid. For materials with a yield stress $\tau_{0}$ like cement paste and concrete, the Bingham or Herschel–Bulkley model is commonly implemented. The material remains rigid below $\tau_{0}$ ($\dot{\gamma }=0\;s^{-1}$) and starts flowing beyond this threshold. However, in computational methods like CFD modeling, this introduces a mathematical discontinuity, as yield stress models lack a defined viscosity at shear rates $\dot{\gamma }=0\;s^{-1}$, leading to numerical instabilities. These instabilities can be regularized through a continuous mathematical definition of the transition from $\dot{\gamma }=0\;s^{-1}$ to $\dot{\gamma }>0\;s^{-1}$. Regularization methods for viscoplastic flow are comprehensively reviewed in [21] and more recently in [30]. Two frequently applied regularization models are the Papanastasiou regularization [31]; see Equation (7) (and similarly, the Bercovier and Engelmann model; see [32]), and the bi-viscous regularization, described by O’Donovan and Tanner in [33] and given in Equation (8):

(7) ηγ˙=τ0(1−e−m γ˙γ˙crit)γ˙+kγ˙n−1

(8) $$\eta (\dot{\gamma })=\min\left\{\begin{array}{c} \eta_{0}\\ \frac{\tau_{0}}{\dot{\gamma }}+k{\dot{\gamma }}^{n-1} \end{array}\right.\;$$

The Papanastasiou regularization in Equation (7) is an exponential blending function defined by the regularization parameter m in-and the critical shear rate ${\dot{\gamma }}_{crit}$ in $s^{-1}$ where the blending function starts. The bi-viscous model introduced in Equation (8) is defined by a plateau value for the viscosity $\eta_{0}$ in Pa∗s. For steady-state flow simulations at high shear rates, the regularization method is mainly required for mathematical stabilization but is not decisive for the numerical result. Frigaard and Nouar, however, stated that the regularization error is the highest close to the yield stress zone [21], which was also shown by Belblidia et al., who stressed the importance of m and $\tau_{0}$ for the results of contraction or expansion flow [34].

A clear proof of concept has yet to be defined for cement and concrete CFD modeling. Both regularization methods have been applied in cement and concrete research, but their effect on the numerical result has yet to be investigated or explained. Gram modeled the slump flow test with the Herschel–Bulkley model and the Papanastasiou regularization [15]. On the contrary, Pereira et al. simulated the slump flow test using the bi-viscous regularization [22]. Schaer et al. used the Herschel–Bulkley model with bi-viscous regularization to simulate Carbopol flow [35]. De Schryver et al. implemented the rheological behavior of thixotropy (time- and shear-dependent viscosity) into the steady-state pipe flow simulation of concrete using the bi-viscous regularization [36]. Two benchmarks analyzed the applicability of CFD for cement and concrete test cases: In [18], the slump flow was simulated in various programs using the Bingham approach without defined regularization or boundary conditions. In [19], both regularization methods were analyzed to simulate steady-state concrete pipe flow but not a stoppage test, where regularization becomes crucial.

The literature review demonstrates that most CFD simulations for cementitious paste flow either simplify the rheological material behavior, investigate steady-state conditions in analytically resolvable geometries, or do not test numerical boundary and regularization methods on their effect on numerical accuracy. Accurate analysis results are generally unavailable, especially for transient test cases published (such as slump flow or L-Box). The effect of spatial-temporal refinements, regularization procedures, boundary conditions, or rheological properties on accuracy and flow patterns needs to be described, and the simulations concentrate on the flow behavior at high shear rates. Recognizing the significant impact that an unadjusted numerical setup can have on the test results, this research addresses this gap by conducting a thorough examination of grid convergence, the geometrical model, regularization parameters, and the transient behavior of cementitious pastes with varying viscosities in the context of the slump flow test.

## 3. Experimental and Numerical Setup

### 3.1. Concept of Investigation

**Figure 1:**
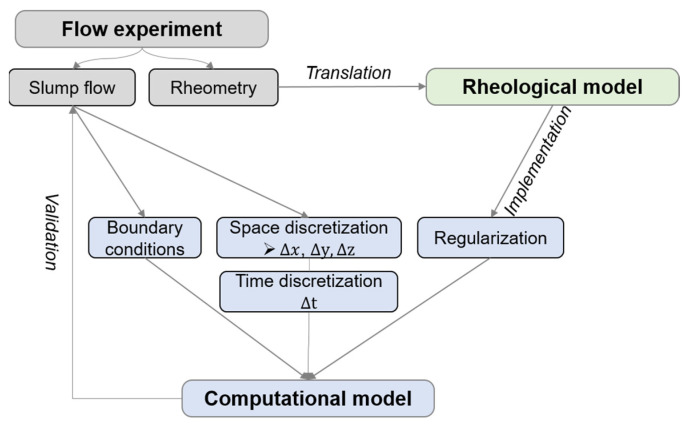
Schematic overview of procedure between experimental flow tests, rheological modeling, and numerical optimization, freely adapted from [40].

Babuska et al. stated, “A computational model relates well to the theory if the computational model describes the mathematical model well and the mathematical model relates to the theory well” [37]. Thus, our approach was three-fold: Experimental flow tests of non-Newtonian pastes were accompanied by rheological measurements. Flow curves gained through rheometry were translated into a rheological model. Flow tests with the slump flow test case were conducted, and the yield stress was analyzed with Equation (1). The computational model described the slump flow test, and the rheological information from rheometric tests were implemented into the computational model. The model was validated computationally by comparison to the experimental flow tests. The whole procedure is illustrated in Figure 1. Definitions as proposed by the AIAA (American Institute of Aeronautics and Astronautics) standards acc. [38] were applied. Detailed explanations on the validation and verification process can be found in [39].

A fully resolved three-dimensional geometrical mesh and a slice geometry using the *wedge* condition of OpenFOAM were compared to use the axisymmetric geometry to save computational cost, but at the same time, with awareness paid to the probable errors. Following the convergence study, the regularization method with the Papanastasiou model in Equation (7) and the bi-viscous model in Equation (8) were tested with varying regularization parameters on the most accurate mesh from the convergence study. Finally, transient flow properties of cementitious pastes with different rheological properties were investigated.

### 3.2. Materials and Experimental Methods

Cement paste was prepared using Ordinary Portland Cement (OPC) CEM I 42.5 R (Heidelberg Materials AG, Heidelberg, Germany), demineralized water with a temperature adjusted for a constant paste temperature of 20 °C and PCE superplasticizer (Master Builder Solutions GmbH, Trostberg, Germany). The oxide composition of the cement is presented in Table 1; physical properties are summarized in Table 2. Further information can be taken from data in brief in [41]. The PCE superplasticizer was provided in a liquid solution with a polymer content of 22.6%. The density was 1.06 kg/L. The anionic charge density was 1614 µeq/g with 20 ethylene oxide units. The anionic charge density was analyzed by Lei et al. using a 0.01 M aqueous NaOH solution at a pH = 12 [42].

Cement pastes with three different solid volume fractions $\Phi_{s}$ were analyzed, with

(9) $$\Phi_{s}=\frac{V_{s}}{V_{s}+V_{w}}$$

In Equation (9), $V_{s}$ is the volume of the solid particles; $V_{w}$ is the volume of the liquid phase. PCE was added to obtain a targeted experimental slump flow diameter 250 mm±5 mm in the Hägermann cone acc. DIN EN 12350-8 [7]/DIN 1015-3 [43] was used for all mixture compositions. The cement paste mixtures of CEM I, demineralized water and PCE, together with the corresponding water-to-cement ratio (w/c) and the resulting paste density $\rho_{p}$, are collected in Table 3. The values for PCE are specified as the percentage by weight of cement (bwoc). Table 3 also shows the analytical $\tau_{0,A,R}$ for the application of Roussel’s Equation (1) for a slump flow diameter of 250 mm (and, thus, a final slump flow radius of R=0.125 m). The values for $\tau_{0,\;A,R}$ only differ due to different input parameters for the paste density $\rho_{p}$. Increasing PCE addition affects the yield stress $\tau_{0}$ and viscosity η [44] and further leads to visco-elastoplastic material behavior rather than viscoplastic flow [45]. However, for simplification reasons, solely viscoplastic modeling was applied.

For each test series, 0.5 L of paste was prepared by mixing water and dry cement using a hand mixer with a four-bladed screw for 90 s at 1700 rpm. PCE was dosed 90 s after mixing had started. The paste was left at rest until 12:30 min after water addition. An external pre-shear of 30 s with the hand mixer led to de-flocculation and erased the effect of structuration history [46]. Rheometric measurements started directly after external pre-shear 15 min after water addition. A dynamic steady shear analysis in a rotational shear rheometric setup was conducted with a parallel plate geometry. Parallel plates with a diameter of 50 mm and serrated surfaces and a small gap of 1 mm were used. A rotational decreasing step-rate protocol from $\dot{\gamma }=80{\;s}^{-1}$ to $\dot{\gamma }=0.02\;s^{-1}$, with each step lasting 6 s, was chosen to grasp the rheological response of cementitious pastes over an extensive range of shear rates $\dot{\gamma }$. Before the step-rate sequence, a pre-shear rate of $\dot{\gamma }=40{\;s}^{-1}$ with a duration of 30 s took place to erase heterogeneous placement effects into the rheometer. A further insight into the rheometrical procedure and raw data handling can be found in [45].

### 3.3. Numerical Setup

The open-source software OpenFOAM (Open-source Field Operation And Manipulation, https://openfoam.org, accessed on 15 December 2023) was used for the solution of the Navier–Stokes equations, which uses Volume-of-Fluid (VoF) method for multiphase flow phenomena to solve the partial differential equations of flow for each spatially discretized volume. In OpenFOAM, air was implemented as first phase and the cementitious paste as the second phase. For each phase, the rheological transport models were specified. The corresponding solver was *interfoam*, which uses the *pimple loop* for the iterative correction of pressure and velocity when solving the transport equations.

#### 3.3.1. Geometrical Model

**Figure 2:**
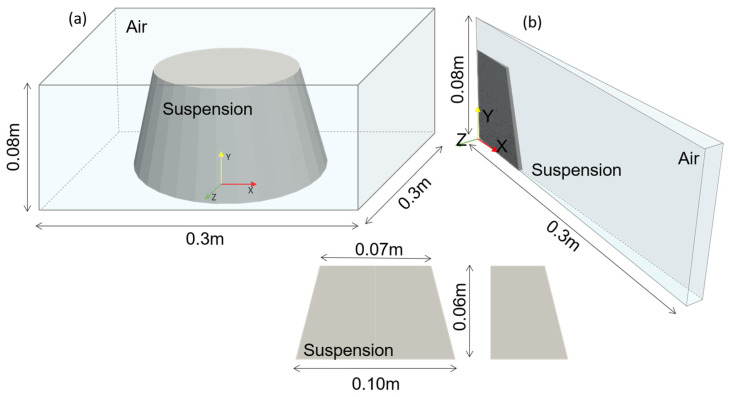
(**a**) Three-dimensional geometry in a box; (**b**) slice geometry in a box.

Figure 2 illustrates the geometrical model. The full three-dimensional resolution of a conical flow (indicated in Figure 2a) depicts the experimental setup. A box was generated with the dimensions 0.3 m×0.3 m×0.08 m that was filled with air (Newtonian rheological model for gas). A cone model was generated with a lower diameter of 0.1 m, an upper diameter of 0.05 m, and a height of 0.06 m, which was placed inside the box. The walls of the Hägermann cone were not modeled. The cone solely represents the cementitious paste. Faces of the geometry were defined either as *ground wall* or as *atmosphere*. The outer box was meshed homogeneously in x, y, and z directions.

Three series with a mesh refinement value of 2 each were generated to study the mesh convergence (see Table 4, C1–C3). The maximum number of cells in the finest mesh refinement series is 7.2 Mio cells. In a second geometrical setup and due to the symmetrical geometry, only a slice of the whole cone was modeled. OpenFOAM enabled the simulation with a slice geometry using a *wedge condition* for the plane faces in the swirling direction for a two-dimensional rotationally symmetric case. In the z direction, only one cell was specified. Thus, at the inner radius r=0 m, cells possessed triangular shapes, and the slice angle was small to delimit the skewness of the cells at the inner and outer radius. At r>0 m, cells were prismatic. Hexahedral cells filled the remaining slice, with increasing aspect ratios along the x direction. An angle of 3° was set for the slice to keep the aspect ratio of cells toward the outer radius close to 1 (see Figure 2b). As the slice setup required fewer cells than the cone geometry, four mesh refinements with a maximum number of cells of 63,441 (with, in this case, 159 prismatic cells and 63,282 hexahedral cells) in the finest mesh setup (see Table 5 and S1–S4) were chosen.

Dimensions of Δx, Δy, and Δz, the number of cells and the mesh refinement value are presented for the three-dimensional cone model in Table 4 and for the slice in Table 5. The aspect ratio of all cone geometries was 1. While the *x*/*y* aspect ratio of slice cells was also defined as 1, the aspect ratio in the slice varied over the radius. The maximum aspect ratio was 2. The effect of the mesh geometry on the simulation result was subsequently investigated.

#### 3.3.2. Boundary Conditions and Numerical Solution Schemes

Boundary conditions for the faces *ground wall* and *atmosphere* for u, p, and α are provided in Table 6. The spatial interpolation was performed with the *linear Upwind method*. Temporal integration was performed with the *implicit Euler method*. Slight under-relaxation was chosen to prevent instabilities. The convergence tolerance for the solver algorithm was set to 1 × 10^−8^. The surface tension between the two phases was defined at $\sigma =0.072\;kg/s^{2}$.

#### 3.3.3. Convergence Study

The Courant number Co calculates how far a fluid moves through one cell depending on the velocity u, the cell size $\Delta x_{i}$, and a defined time step Δt:

(10) $$Co=\frac{u\Delta t}{\Delta x_{i}}\;$$

If Co<1, the fluid information does not propagate through more than one cell within one time step. If Co>1, the simulation is inaccurate. Thus, for many simulation cases, Co was set to a limit value $Co_{max}$.

In the numerical setup, $Co_{max}=1$ was defined to prevent numerical instabilities. However, as the slump flow case is a transient process where both the velocity and the slump shape change with every single time step, a characteristic Co does not exist for the whole test case. Locally varying Co over the shape, along with the effect of Δx and Δt, and, thus, the effect of the actual $Co_{c,max}$ on the simulation result was investigated. Convergence was studied threefold:

- *Grid convergence*: The grid convergence study analyzed flow results as a function of increasing grid refinement at a fixed time step Δ*t*. For the 3D geometry, three meshes (Δx=0.004, 0.002 and 0.001) were tested for convergence. Different fixed time steps of $\Delta t=1\times 10^{-3}$ s, $5\times 10^{-4}$ s, and $2.5\times 10^{-4}$ s were studied. For the slice geometry, four meshes (Δx=0.004 m, 0.002 m, 0.001 m, and 0.0005 m) were tested at fixed $\Delta t=5\times 10^{-4}\;s$, $2.5\times 10^{-4}$ s, and $1.25\times 10^{-4}$ s.
- *Temporal convergence*: Temporal convergence was tested for each mesh (C1–C3 and S1–S4) with fixed Δx for five time steps Δt, respectively (see the range of Δt for each fixed Δx in Table 7).
- *Coupled spatial and temporal convergence*: Simulations with coupled spatial and temporal refinement were tested for convergence. For the 3D geometry, three setups were tested; for the slice geometry (due to higher possible spatial refinement), five series were compared (see Table 7: Convergence series in the diagonal with the same color)

The post-processing procedure of the simulated data was conducted using the freeware Paraview (https://www.paraview.org/, accessed on 15 December 2023) and python (https://www.python.org/, accessed on 15 December 2023). The simulations covered a real flow time between $0\;s<t_{of}<2\;s$. The time of flow $t_{of}$ in s was defined at a threshold where the slump flow radius did not deviate more than $5e^{-3}\%$ compared to the flow of the previous time step. Output data were generated every 0.02 s between 0 s and 0.4 s and every 0.2 s between 0.4 s and 2.0 s.

Numerical results were compared to the experimental flow test result. The percentage deviation of the numerical to the experimental slump flow radius $r_{x}$ was calculated as error $e_{x}$ in %. The convergence study was only conducted with the rheological input data of the test series OPC-0.45.

#### 3.3.4. Regularization Study

For the slump flow test, the definition of the regularization model becomes crucial: the slump flow test both starts and ends with no flow ($\dot{\gamma }=0$), and, thus, a mathematically undefined state.

The Papanastasiou and bi-viscous regularization were applied to the Herschel–Bulkley model for non-Newtonian yield stress fluids as introduced in Equations (7) and (8). Following the convergence study, the regularization test was solely conducted for the test series OPC-0.45 and with the most accurate geometrical mesh. The regularization parameters are presented in Table 8. In the literature, the critical shear rate to be implemented into the Papanastasiou model is either proposed to be set to a minimum value or is not mentioned at all (see, e.g., [18]). In a comparative approach, the critical shear rate ${\dot{\gamma }}_{crit}$ was varied to investigate the effect of the calibration of the model by its defined critical shear rate. In a first approach, ${\dot{\gamma }}_{crit}$ was defined at a low value, with ${\dot{\gamma }}_{crit}=0.001\;s^{-1}$. In a second step, the critical shear rate ${\dot{\gamma }}_{crit}$ was evaluated from the rheological experiment, subsequently elaborated in Section 4.1. For the test series OPC-0.45, this yielded ${\dot{\gamma }}_{crit}=0.16\;s^{-1}$. Simulations were conducted with each ${\dot{\gamma }}_{crit}$, respectively, for each m.

**Figure 3:**
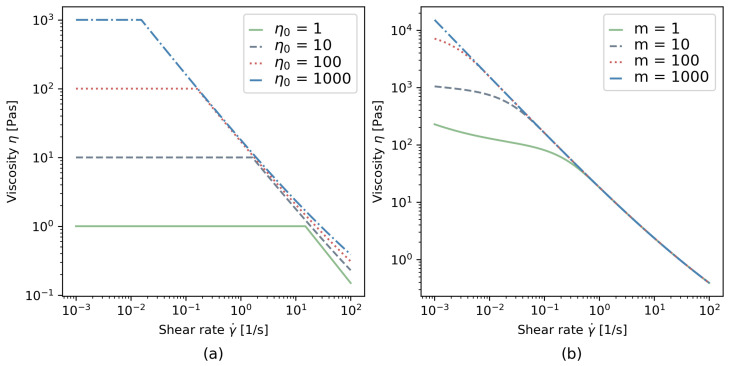
Computed viscosity curves (**a**) for the bi-viscous model and (**b**) for the Papanastasiou model.

For the bi-viscosity model, four different zero viscosities $\eta_{0}$ were defined, i.e., $\eta_{0}=1,\;10,\;100$, and 1000 Pa∗s (for more details of rheological calculation and material discontinuities, please refer to the author’s publication [45]). The zero-shear rate ${\dot{\gamma }}_{0}$ was calculated depending on the condition in Equation (7). An illustration of the rheological models is presented in Figure 3.

## 4. Results and Discussion

### 4.1. Rheological Analysis

**Figure 4:**
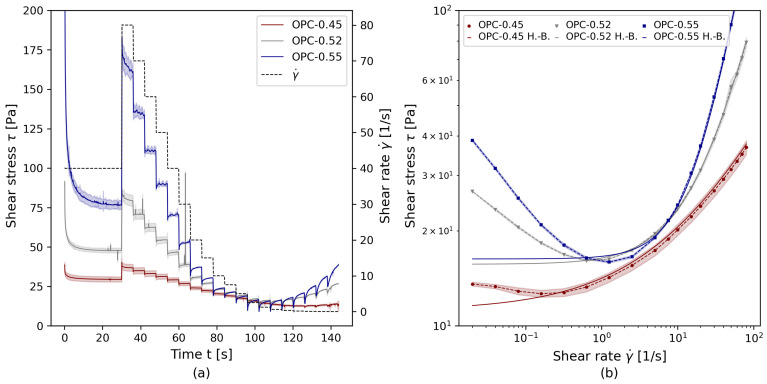
Rheological data for the test series OPC-0.45, OPC-0.52 and OPC-0.55: (**a**) rheological step-rate dynamic rotational shear experiment, (**b**) $\dot{\gamma }-\tau$ flow curves.

Experimental raw data and the corresponding rheological flow curves of all test series are illustrated in Figure 4. Figure 4a shows the average values, incl. standard deviation (shades) for the measured shear stress as a function of the shear rate $\dot{\gamma }$. Each paste was analyzed at least three times. The experimental data show an equilibrium stress τ after the pre-shear at $\dot{\gamma }=40\;s^{-1}$ within the first 30 s. With the exception of the first shear rate step at $\dot{\gamma }=80\;s^{-1}$, τ reached equilibrium at each $\dot{\gamma }$—step before increasing again at low shear rates. The minimum stress measured before the stress increase at low $\dot{\gamma }$ was defined as ${\dot{\gamma }}_{crit}$. From each $\dot{\gamma }$—step, the shear stress τ was calculated at equilibrium.

Figure 4b shows the calculated $\tau -\dot{\gamma }$ flow curves. The log-log illustration was chosen to highlight both the strong deviation of the stress response at low shear rates while also clearly depicting the deviating rheological behavior at high shear rates. At higher shear rates, shear-thickening behavior increases with increasing solid volume fraction. To calculate rheological parameters, the Herschel–Bulkley regression was chosen for the experimental data in the range between [${\dot{\gamma }}_{crit}\;\leq \dot{\gamma }\leq \;{\dot{\gamma }}_{max}$], with ${\dot{\gamma }}_{crit}$ as the shear rate at a minimum shear stress τ. Critical shear rates ${\dot{\gamma }}_{crit}$ increased with increasing solid volume fractions of the pastes; see Table 9. The Herschel–Bulkley regression parameters, ${\dot{\gamma }}_{crit}$, and the corresponding computational kinematic input parameters, which are the rheological parameters divided by the paste density $\rho_{p}$, are collected in Table 9. While OPC-0.45 showed shear-thinning material behavior, OPC-0.52 was close to a linear viscosity with n=1, and OPC-0.55 was strongly shear-thickening with n=1.41.

The results show that, while the targeted PCE ensured comparable slump flow diameters and, thus, comparable macroscopic flowability, the rheological properties of the different pastes strongly deviated depending on solid volume fraction $\phi_{s}$.

### 4.2. Numerical Model Analysis

#### 4.2.1. Post-Processing Strategy: Transient Flow Data Extraction

**Figure 5:**
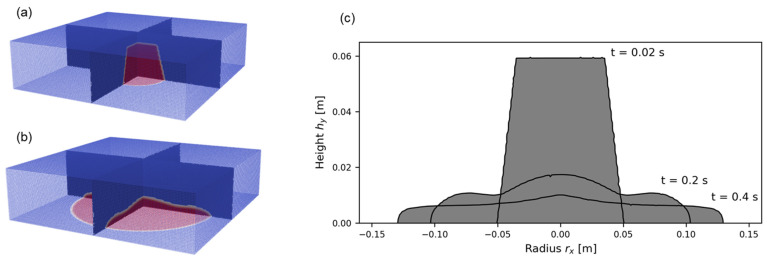
Post-processing in Paraview for (**a**) *t* = 0 s and (**b**) *t* = 1 s of flow, (**c**) two-dimensional extracted flow data for *t* = 0.02 s, 0.2 s and 0.4 s with post-processing in Python.

**Figure 6:**
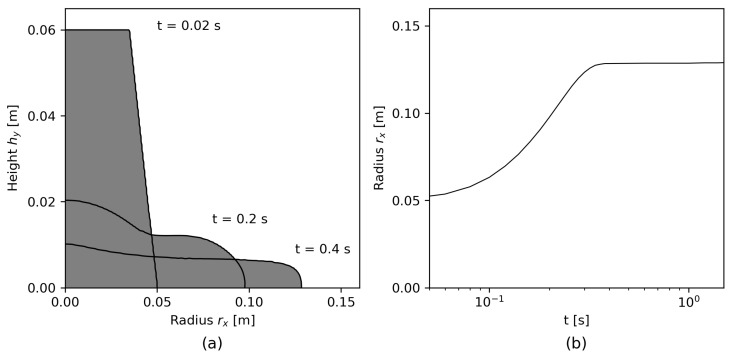
Schematic flow analysis of a case simulation: (**a**) two-dimensional plot of sample shapes for three different time steps, (**b**) one-dimensional plot for the slump flow radius $r_{x}$ over time.

**Figure 7:**
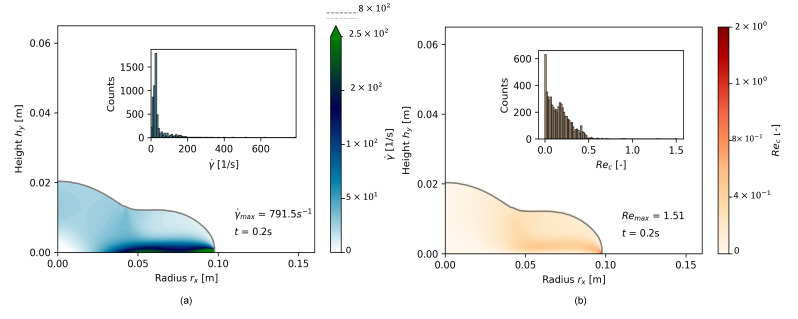
Transient flow information for two-dimensional slice data for (**a**)$\;\dot{\gamma }$ and (**b**) $Re_{c}$.

Each numerical flow calculation was initially post-processed in Paraview. Figure 5, Figure 6 and Figure 7 illustrate the post-processing on the example of the test series OPC-0.45.

Figure 5a,b show the three-dimensional slump flow at a time of flow of 0 s and 1 s, respectively.

From the three-dimensional raw data, a two-dimensional slice was extracted. Figure 5c illustrates the slump flow for three time steps (t=0.02 s, t=0.2 s, t=0.4 s) in python. In all subsequent procedures and calculations, considering the symmetry of the flow, only one symmetric part was analyzed, illustrated in Figure 6a. The evolution of the flow radius over time $r_{x}(t)$ is presented in Figure 6b.

In addition to the shape information, the slump flow radius $r_{x}(t)$ and height $h_{y}\left(t\right)$), the cell Reynolds number $Re_{c}$, and the shear rate $\dot{\gamma }$ were extracted and analyzed for each time step. The Reynolds number Re is a characteristic dimensionless flow number, which relates the inertial forces to viscous forces:

(11) $$Re=\frac{\rho uL}{\mu }$$

With ρ as the fluid density in $kg/m^{3}$, u as the fluid velocity in $\frac{m}{s}$, L as the characteristic length scale in m, and μ as the fluid viscosity in Pa∗s. Re requires the definition of a characteristic length scale L, characteristic velocity u, and characteristic viscosity μ. In this study, due to a missing characteristic length scale, the cell Reynolds number $Re_{c}$ is introduced, which calculates $Re_{c}$ according to Equation (11), using the cell velocity u, Δx and Δt, and the paste density $\rho_{p}$.

Figure 7 shows the two-dimensional data analysis of $Re_{c}$ and $\dot{\gamma }$ for t=0.2 s for the finest slice mesh S4 with $\Delta x=5\times 10^{-4}\;m;\Delta t=3.125\times 10^{-5}\;s$. A color bar illustrates the variation of rheological properties over the two-dimensional shape. The density distributions of $Re_{c}$ and $\dot{\gamma }$, respectively, are illustrated in an additional histogram. The histogram had a bin value of 100, and the number of counts was calculated with regard to their distribution over the cells of the two-dimensional slice. The illustration shows the locally different flow patterns over the shape: while after 0.2 s, the flow had already stopped around $x_{r}=0\;m,\;h_{y}=0\;m$, high $\dot{\gamma }$ and, thus, high $Re_{c}$ occur toward the maximum $x_{r}$ and a thin layer close to the bottom of the slump, with the maximum values specified in Figure 7.

#### 4.2.2. Convergence Study

**Figure 8:**
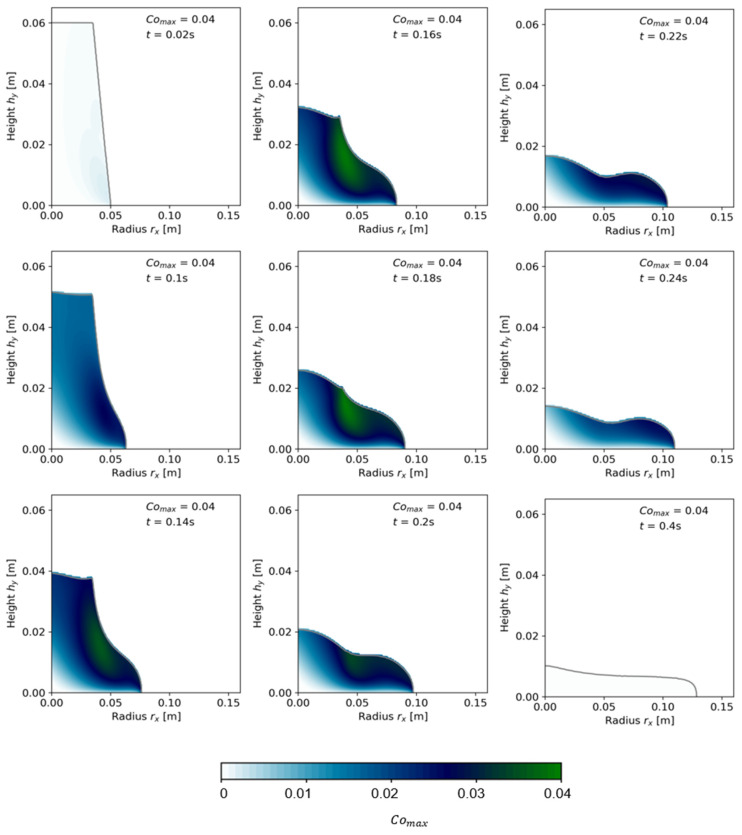
Plot of Courant numbers with calculation of the maximum Courant numbers $Co_{max}$ for various time steps from start of flow until rest; here, the example with the finest mesh resolution S4, $\Delta x=5\times 10^{-4}\;m;\Delta t=3.125\times 10^{-5}\;s$ for 9 selected time steps is shown.

The convergence and regularization study were conducted on a cement paste with $\Phi_{s}$ = 0.45; see Table 3, test series OPC-0.45. After the setup of an accurate geometrical model and regularization model, the effect of increasing viscosity on transient flow modeling was investigated with additional cementitious suspensions with $\Phi_{s}$ = 0.52 and with $\Phi_{s}$ = 0.55; see Table 3, test series OPC-0.52 and OPC-0.55, respectively. For the grid convergence study, $Co_{max}$ was recorded for each time step, as schematically illustrated in Figure 8 with the depiction of Courant numbers Co for each cell over the two-dimensional shape.

**Figure 9:**
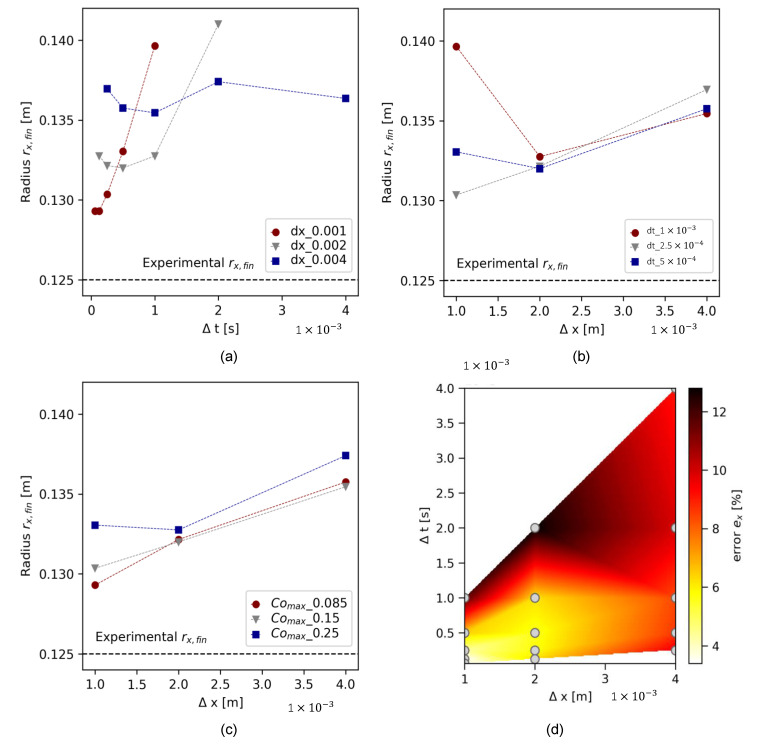
Convergence analysis results for 3D cone geometry for (**a**) temporal refinement, (**b**) spatial refinement, (**c**) aligned Δx and Δt with refined Courant numbers, and (**d**) error plot for all simulations at different pairs Δx, Δt.

**Figure 10:**
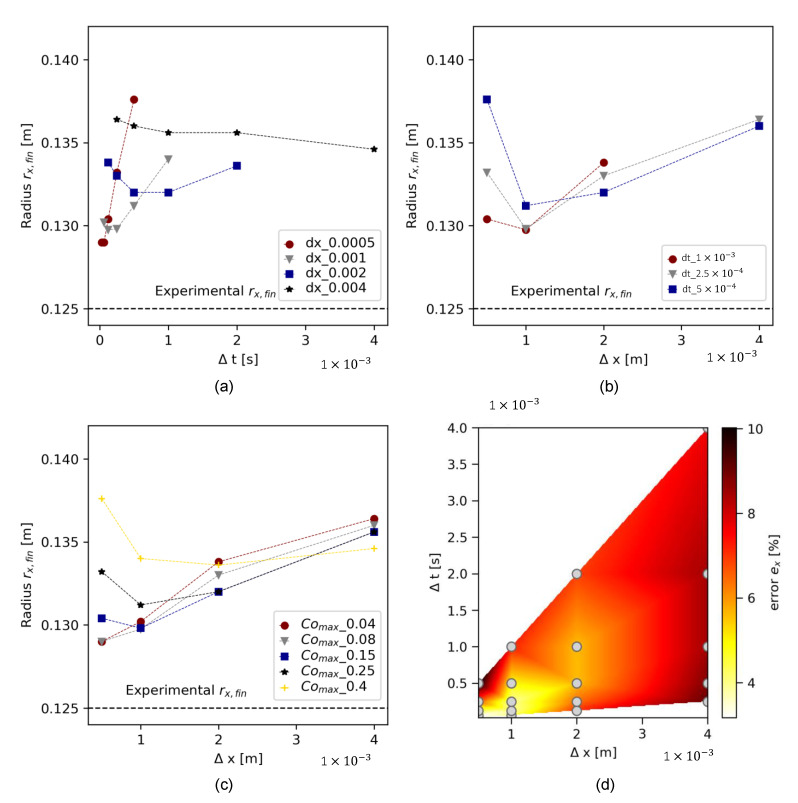
Convergence analysis results for slice geometry for (**a**) temporal refinement, (**b**) spatial refinement, (**c**) aligned Δx and Δt for refined $Co_{max}$, and (**d**) error plot for all simulations at different pairs Δx, Δt.

Convergence study results are demonstrated in Figure 9 for the cone geometry and in Figure 10 for the slice geometry. Each figure shows the experimental flow result as a dotted line at $r_{x,fin}=0.125\;m$. In both illustrations, (a) presents the final flow radius $r_{x}$ as a function of Δt for different meshes (temporal refinement), (b) presents the slump flow radius as a function of Δx for various time step sizes, and (c) shows the slump flow radius for simulations where both Δx and Δt were refined. The simultaneous refinement of Δx and Δt resulted in similar, decreasing maximum Courant numbers $Co_{max}$ = 0.25, 0.15, and 0.085 for the three-dimensional mesh and $Co_{max}=0.4,\;0.25,\;0.15,\;0.08$, and 0.04 for the slice. Figure 9d and Figure 10d illustrate the percentage error $e_{x}$ of the simulated slump flow radius related to the experimental final slump flow radius $r_{x,fin}$:

(12) $$e_{x}=\frac{r_{x,fin,\;num}}{r_{x,fin,\;exp}}*100$$

Figure 9a,b indicate that the simulated final slump flow radius $r_{x,fin}$ converged toward the experimentally measured values as refinement values increased up to a certain threshold. With further refinement, the error between numerical and experimentally measured values increased. Figure 9c illustrates the correlation between Δx and Δt: if the ratio between Δx and Δt is kept constant, the error decreases with increasing refinement rate, at least at Co<0.25. For $Co_{max}=0.25$, the simulation error again increased below Δx=0.002 m. For the lowest $Co_{max}=0.085$, the error was decreased to a minimum of about $e_{x}\approx 4\%$; see Figure 9d. Figure 10 presents comparable results for the slice geometry.

Due to a smaller geometry and thus less cells, higher refinement values were possible. The effect of temporal refinement on a fixed mesh grid, however, was more pronounced for the slice than for the cone. The finest mesh S4 showed the highest variation of $r_{x}$ in dependence of Δt. Figure 10c shows, similarly to the cone geometry, that mesh convergence was only reached at $Co_{max}\leq 0.08$. The results reveal the strong dependency of both temporal and spatial refinement on the numerical result.

#### 4.2.3. Comparison between Cone and Slice Simulations

**Figure 11:**
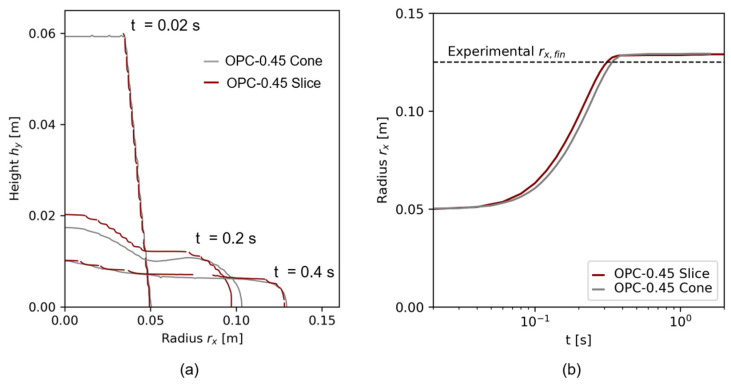
Comparison of 3D and slice geometry for the fines spatial and temporal resolution, each (**a**) exemplary shape comparison for t=0.02 s and t=0.38 s, (**b**) flow over time.

Shapes for the flow times 0.02 s,0.2 s, and 0.4 s for the finest cone mesh C3 and the finest slice mesh S4 are illustrated in Figure 11a, and the slump flow radius $r_{x}$ in dependence of t is given in Figure 11b. Slight variations were visible during the time of flow, as the cone geometry flowed faster and thus had a larger $r_{x}$ (and lower height $h_{y}$) during the same time step. The final slump flow radius, however, was the same. Figure 11a illustrates a clear shape difference between the cone and the slice model at the time step 0.2 s. This result can be correlated to the mesh conditions: At r=0, the cone geometry possessed hexahedral cells, while the slice geometry consisted of prisms. The aspect ratio between the cone cells (aspect ratio = 1) and the slice cells at r=0 (aspect ratio = 2) differed. A computational effect on the flow therefore was inevitable. A further analysis of numerical effects of the aspect ratio on the numerical result is beyond the scope of this research but must be considered for the further investigations.

The mesh comparison shows that slight deviations existed between the three-dimensional cone and a slice geometry with the *wedge* condition provided by OpenFOAM. However, the wedge geometry was used for further analysis due to reduced computational costs.

### 4.3. Effect of Regularization Parameters on Numerical Simulation

**Figure 12:**
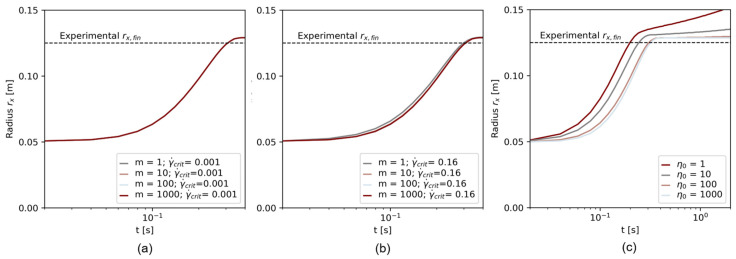
Results of the regularization study for (**a**) the Papanastasiou regularization with $\dot{\gamma }=0.001\;s^{-1}$, (**b**) the Papanastasiou regularization with $\dot{\gamma }=0.16\;s^{-1}$, and (**c**) the bi-viscosity model.

Figure 12 shows the flow over time for varying regularization parameters. Figure 12a,b show the Papanastasiou model with (a) ${\dot{\gamma }}_{crit}=0.001\;s^{-1}$ and (b) ${\dot{\gamma }}_{crit}=0.16\;s^{-1}$. Once a minimum shear rate of ${\dot{\gamma }}_{crit}=0.001\;s^{-1}$ was chosen, the regularization parameter m did not affect the slump flow radius at all, no difference between the simulation results is observable, see Figure 12a. At higher ${\dot{\gamma }}_{crit}$, m can slightly affect the flow progress, as visible in Figure 12b: At low m=1, the flow progressed faster than at higher m values. In Figure 12c, the flow with the bi-viscous regularization is illustrated. With decreasing $\eta_{0}$, the flow velocity (slope of the curve $r_{x}(t)$) was higher, and the final slump flow radius increased. The parameters $\eta_{0}=1$ and $\eta_{0}=10$ did not show a flow stoppage. With increasing $\eta_{0}$, the error to the experimental result decreased.

The results of the regularization study prove the applicability of the Papanastasiou regularization method for a transient flow simulation that includes the start of flow and flow stoppage. However, also when using the Papanastasiou regularization method, the parameters ${\dot{\gamma }}_{crit}$ and m affect the numerical result. While m displays the mathematical regularization, which cannot be connected to real rheological flow behavior, ${\dot{\gamma }}_{crit}$ has a rheological meaning. Therefore, the choice of which ${\dot{\gamma }}_{crit}$ to use for an accurate flow simulation becomes more crucial with increasing ${\dot{\gamma }}_{crit}$.

### 4.4. Numerical Flow of Different Viscous Cementitious Pastes

#### 4.4.1. Regularization and Flow over Time

Finally, the flow of different cementitious pastes with increasing $\Phi_{S}$ was investigated numerically. The calculated critical shear rates ${\dot{\gamma }}_{crit}$ from the rheometric experiments were ${\dot{\gamma }}_{crit}=0.64\;s^{-1}$ for OPC-0.52 and ${\dot{\gamma }}_{crit}=1.25\;s^{-1}$ for OPC-0.55.

**Figure 13:**
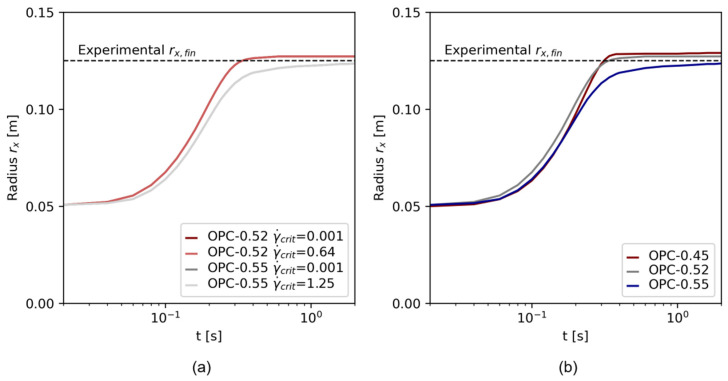
Flow over time $r_{x}(t)$ for (**a**) OPC-0.52 and OPC-0.55 with different regularization parameters and (**b**) flow over time $r_{x}(t)$ for OPC-0.45, OPC-0.52 and OPC-0.55.

For the Papanastasiou regularization, they were implemented as ${\dot{\gamma }}_{crit}$. Comparatively, also ${\dot{\gamma }}_{crit}=0.001\;s^{-1}$ was tested; see Table 10. The regularization parameter m was fixed at m=1000 for all simulations. Figure 13a illustrates the flow simulations specified in Table 10. No variation was visible for different ${\dot{\gamma }}_{crit}$ as m was chosen to be high. Thus, results for OPC-0.52 and OPC-0.55 (here with the experimental ${\dot{\gamma }}_{crit}$) were compared to the flow results of OPC-0.45. The flow over time $r_{x}(t)$ for all test series is presented in Figure 13b.

The analysis reveals that the rheological behavior, specified by the Herschel–Bulkley model, strongly affected the time-dependent flow. OPC-0.45 approached its final slump value after 0.5 s of flow, while the flow velocity decreased with the increasing solid volume fraction. Interestingly, OPC-0.52 proceeded faster in the beginning of the test, which can be attributed to a higher paste density $\rho_{s}$ and, thus, a higher hydrostatic stress tensor. However, due to a higher viscosity, the flow proceeded slower compared to OPC-0.45. Test series OPC-0.55 with the highest viscosity and shear-thickening flow behavior showed the slowest flow progress. The simulation time of 2.0 s was not sufficient to analyze the flow stoppage. The numerical results are provided in Table 11. In addition to the final slump flow radius $r_{x}$ and the time of flow, the maximum cell values for the shear rate ${\dot{\gamma }}_{max,c}$, the cell Reynolds number $Re_{max,c}$ and Courant number $Co_{max}$ are specified. For a deeper understanding of transient flow differences depending on the paste’s viscosity, the cell Reynolds number $Re_{c}$ and the shear rate $\dot{\gamma }$ were analyzed over the two-dimensional shape.

#### 4.4.2. Transient Flow Patterns

**Figure 14:**
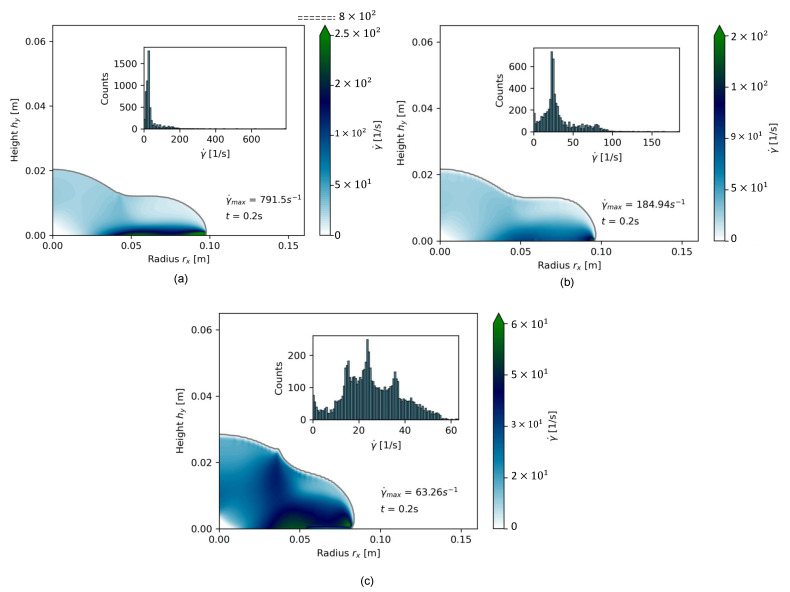
Comparative shear rate distribution for (**a**) OPC-0.45, (**b**) OPC-0.52, and (**c**) OPC-0.55 for 0.2 s of flow time.

Figure 14 illustrates the shear rate distribution $\dot{\gamma }$ for all pastes OPC-0.45, OPC-0.52, and OPC-0.55 for a time step of t=0.02 s. Table 11 and Figure 14 clearly show a paste-dependent difference of ${\dot{\gamma }}_{max}$ and the $\dot{\gamma }$-distribution over the slump’s shape: The highest shear rate value for OPC-0.45 was $791.5\;s^{-1}$. The number of high shear rates was higher than for the other pastes. OPC-0.52 had a highest $\dot{\gamma }$-value of ${163.08\;s}^{-1}$, while most shear rates at t=0.02 s were between $0\;s^{-1}<\dot{\gamma }<50\;s^{-1}$. OPC-0.55 had the highest ${\dot{\gamma }}_{max}$ $=63.26\;s^{-1}$.

**Figure 15:**
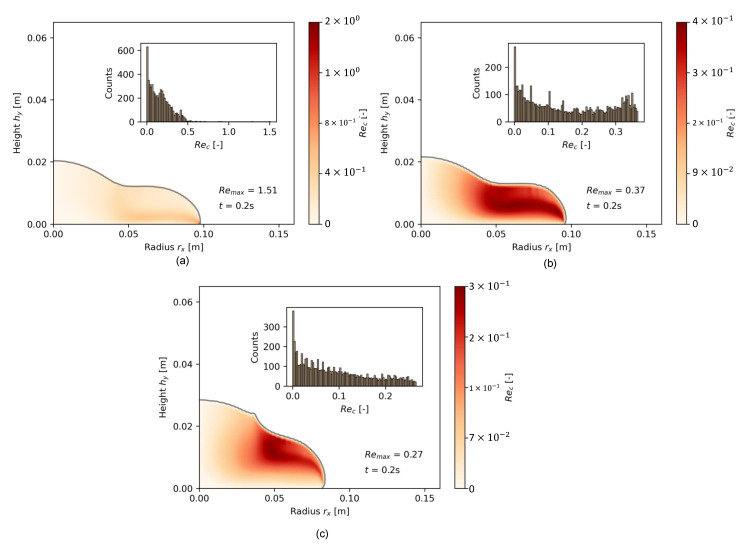
Comparative distribution of the cell Reynolds number for (**a**) OPC-0.45, (**b**) OPC-0.52, and (**c**) OPC-0.55 for 0.2 s of flow.

Figure 14c further shows that the shear rate distribution within one time step, here exemplarily for t=0.02 s, was much more diverse. Instead of a strongly skewed log-normal distribution for a fast-flowing, low-packed cement paste, the shear rate distribution became chaotic once a cementitious paste was tested that diverged from low, shear-thinning viscosities. In Figure 15, the same comparison is given for the cell Reynolds number $Re_{c}$. With increasing $\Phi_{s},\;Re_{c}$ decreased strongly as the cell velocity decreased. Again, OPC-0.52 and OPC-0.55 possessed a wider density distribution of $Re_{c}$ compared to OPC-0.45.

The shape plots and density distribution provide insights into the transient flow properties during the flow of different viscous pastes. Cementitious pastes with low viscosity quickly approached the final slump flow radius, with the velocity gradient predominantly directed toward the outermost region of the paste. In contrast, the flow occurred more slowly in pastes with a higher solid volume fraction $\Phi_{s}$, and consequently, a higher apparent viscosity η. In such cases, a wide range of flow states became apparent across the flow body.

## 5. Conclusions

The study reveals critical insights into the influence of the numerical setup on the accuracy of simulating viscous cementitious paste slump flow, and followed by this, transient flow phenomena of different viscous pastes. The main conclusions are:

- The convergence study showed a significant combined spatial and temporal discretization effect on the final flow result. Co < 0.1 provided numerical errors at around 4% compared to real-life scenarios.
- The slice model provided a high numerical accuracy at Co≤0.08 with errors $e_{x}<4\%$. The spatial-temporal refinement, however, affected the numerical result more than the cone geometry.
- Regularization affected the numerical slump flow radius. The bi-viscous regularization led to varying numerical results depending on $\eta_{0}$. The Papanastasiou regularization led to a decreased effect of numerical regularization on the final flow result at m ≥1000. A final question is posed: Is it meaningful for all cementitious pastes to fix regularization parameters at a high value to decrease their effect on the final slump flow radius? Or could the regularization parameters present the real rheological behavior approaching resting conditions? The choice of high $\eta_{0}$ or high m seems feasible to not manipulate the Herschel–Bulkley model. However, adapted rheological models that specify rheological paste properties at slow flow, in combination with mathematical regularization methods, could lead to simulation results that depict flow phenomena that are physically correct.
- The analysis of transient flow patterns in the two-dimensional slump shape revealed the wide range of rheological properties during a single time step. This aids in understanding the non-Newtonian flow behavior of cementitious pastes and enables the analysis of time-dependent rheological phenomena.

To summarize, the findings serve as a basis for further rheological parameter studies and to investigate the slow flow phenomena of cementitious pastes more closely. Since the apparent viscosity is affected by the shear rate, cementitious paste exhibits varying viscosity values across its shape. This aspect becomes significant when investigating time-dependent flow cases and the impact of rheological parameters on the evolution of transient flow.

Further studies could develop distinct characteristic numbers to describe the non-Newtonian flow evolution. The time-dependent shape analysis could relate low Reynolds numbers to viscous flow effects. While the experimental slump flow test has been an efficient method to characterize the rheological properties of cementitious pastes, the presented CFD setup also provides a numerical slump flow test that supports the evaluation of transient paste properties. This method of investigation can be upscaled to real cement and concrete flow processing computations, such as the time-dependent flow of Self-Compacting concrete (SCC), the analysis of highly viscous flow of Ultra-High-Performance Concrete (UHPC) and the investigation of transient properties of various concretes with strongly differing non-Newtonian behavior during mixing and pumping. Computational modeling then can help to target flow properties and optimize the processing setup. Prospectively, the effect of time- and shear-rate-dependent thixotropy and other rheological models on the flow evolution of cementitious paste and building materials will be investigated. Accurate CFD modeling, incorporating extended rheological models, will finally enhance our understanding of the transient non-Newtonian flow of cementitious building materials beyond what was previously possible.

## Figures and Tables

**Table 1:** Oxide composition of CEM I 42.5 R.

Binder	CaO	SiO_2_	Al_2_O_3_	Fe_2_O_3_	MgO	Na_2_O	K_2_O
	[%]	[%]	[%]	[%]	[%]	[%]	[%]
CEM I 42.5 R (OPC)	62.90	19.63	5.23	2.60	1.54	0.24	0.80

**Table 2:** Physical and granulometric properties of CEM I 42.5 R.

Binder	$\mathrm{Specific}\;\mathrm{Gravity}\;\rho_{c}$	Blaine SSA	d_50_	$\Phi_{max}$
	$\left[g/cm^{2}\right]$	$\left[cm^{2}/g\right]$	[µm]	[−]
CEM I 42.5 R (OPC)	3.11	3499	15.0	0.66

**Table 3:** Cement paste mixtures.

Mixture	$\phi_{s}$	w/c Ratio	Slump Flow Diameter	CEM I	Demineralized Water	PCE	$\rho_{p}$	$\tau_{0A,R}$
	[−]	[−]	[mm]	$\left[kg/m^{3}\right]$	$\left[kg/m^{3}\right]$	[% bwoc]	$\left[kg/m^{3}\right]$	[Pa]
OPC-0.45	0.45	0.40	250 ± 5	1399.5	550	0.18	1950	12.0
OPC-0.52	0.52	0.29	250 ± 5	1617.2	480	0.85	2100	14.2
OPC-0.55	0.55	0.26	250 ± 5	1710.5	450	1.40	2160	15.0

**Table 4:** Mesh definition for the 3D cone (C).

Test Series	Δx	Δy	Δz	Aspect Ratio *x*/*y*/*z*	ΣCells	Mesh Refinement Value
	[m]	[m]	[m]	[−]	[−]	[−]
C1	0.004	0.004	0.004	1	112,500	1
C2	0.002	0.002	0.002	1	900,000	2
C3	0.001	0.001	0.001	1	7,200,000	4

**Table 5:** Mesh definition for slice geometry (S).

Test Series	Δx	Δy	Slice Angle	Aspect Ratio *x*/*y*	ΣCells	Mesh Refinement Value
	[m]	[m]	[°]	[−]	[−]	[−]
S1	0.004	0.004	3	1	931	1
S2	0.002	0.002	3	1	3861	2
S3	0.001	0.001	3	1	15,721	4
S4	0.0005	0.0005	3	1	63,441	8

**Table 6:** Numerical boundary conditions.

Field	Face	Type	Definition	Value
Fluid α	Ground wall	Zero gradient	Neumann	$\frac{\partial }{\partial t}=0$
	Atmosphere	Zero gradient	Neumann	∇α=0
Pressure p	Ground wall	Fixed flux	Neumann	∇p=0
	Atmosphere	Total value	Dirichlet	p=0
Velocity u	Ground wall	No slip	Dirichlet	u=0
	Atmosphere	Inletoutlet	Neumann	∇·u=0

**Table 7:** Test series and the corresponding time step Δt and mesh size Δx.

Test Series/Δt	$4\times 10^{-3}$	$2\times 10^{-3}$	$1\times 10^{-3}$	$5\times 10^{-4}$	$2.5\times 10^{-4}$	$1.25\times 10^{-4}$	$6.25\times 10^{-5}$	$3.125\times 10^{-5}$
C1 $\Delta x=4\times 10^{-3}$	x	x	x	x	x			
C2 $\Delta x=2\times 10^{-3}$		x	x	x	x	x		
C3 $\Delta x=1\times 10^{-3}$			x	x	x	x	x	
S1 $\Delta x=4\times 10^{-3}$	x	x	x	x	x			
S2 $\Delta x=2\times 10^{-3}$		x	x	x	x	x		
S3 $\Delta x=1\times 10^{-3}$			x	x	x	x	x	
S4 $\Delta x=5\times 10^{-3}$				x	x	x	x	x

**Table 8:** Regularization parameters for Papanastasiou and bi-viscous regularization.

Test Series	Papanastasiou		Bi-Viscous	
	m	${\dot{\gamma }}_{crit}$	$\eta_{0}$	${\dot{\gamma }}_{0}$
	[−]	[1/s]	[Pa∗s]	[1/s]
R1	1	0.001/0.16	1	15.1
R2	10	0.001/0.16	10	1.64
R3	100	0.001/0.16	100	0.16
R4	1000	0.001/0.16	1000	0.016

**Table 9:** Rheological parameters from experimental measurements and kinematic input parameters for computational simulations.

Test Series	Herschel-Bulkley Regression				Computational Input		
	${\dot{\gamma }}_{crit}$	${\tau_{0,H-B}}_{exp}$	$k_{exp}$	$n_{exp}$	$\tau_{0,H-B_{num}}$	$k_{num}$	$n_{num}$
	[1/s]	[Pa]	$\left[{\left(Pa*s\right)}^{n}\right]$	[−]	$[m^{2}/s^{2}]$	$[m^{2}/s]$	[−]
OPC-0.45	0.16	11.1	2.86	0.45	5.69 × 10^−3^	2.56 × 10^−3^	0.45
OPC-0.52	0.64	15.3	0.83	0.99	7.31 × 10^−3^	3.95 × 10^−4^	0.99
OPC-0.55	1.25	16.4	0.29	1.41	7.65 × 10^−3^	1.34 × 10^−4^	1.41

**Table 10:** Regularization parameters for the Papanastasiou regularization.

Test Series	Papanastasiou	
	m	${\dot{\gamma }}_{crit}$
	[-]	[1/s]
OPC-0.52-R1	1000	0.001
OPC-0.52-R2	1000	0.640
OPC-0.55-R1	1000	0.001
OPC-0.55-R2	1000	1.250

**Table 11:** Specified flow data.

Series	$r_{x}$	Time of Flow	${\dot{\gamma }}_{max,c}$	$Re_{c,max}$	$Co_{max}$
	[m]	[s]	[1/s]	[-]	[-]
OPC-0.45	0.129	0.5	863.9	1.65	0.04
OPC-0.52	0.127	0.8	175.7	0.43	0.04
OPC-0.55	0.122	>2.0	64.5	0.27	0.035

## Data Availability

The data presented in this study are available on request from the corresponding author.
